# Infrared neuroglial modulation of spinal locomotor networks

**DOI:** 10.1038/s41598-024-73577-4

**Published:** 2024-09-27

**Authors:** Nathan Dumas, Emilie Pecchi, Rodney O’Connor, Rémi Bos, David Moreau

**Affiliations:** 1 Mines Saint-Etienne, Centre CMP, Département BEL, 13541 Gardanne, France; 2 Institut de Neurosciences de la Timone, CNRS UMR 7289 et Aix- Marseille Université, 13005 Marseille, France

**Keywords:** Cellular neuroscience, Astrocyte, Biomedical engineering, Ca2+ imaging, Multiphoton microscopy

## Abstract

Infrared neural stimulation (INS) emerges as a promising tool for stimulating the nervous system by its high spatial precision and absence of the use of exogenous agents into the tissue, which led to the first successful proof of concept in human brain. While neural networks have been the focal point of INS research, this technique is also non cell type specific as it triggers activity in non electrically excitable cells. Despite increasing interest, there remains to demonstrate well defined simultaneous astrocytic and neuronal signals in response to INS. Using calcium imaging, we show that INS has the capacity to initiate calcium signaling in both astrocytes and neurons simultaneously from the rostral lumbar spinal cord, each exhibiting distinct temporal and amplitude characteristics. Importantly, the mechanism underlying infrared-induced neuronal and astrocytic calcium signaling differ, with neuronal activity relying on sodium channels, whereas induced astrocytic signaling is predominantly influenced by extracellular calcium and TRPV4 channels. Furthermore, our findings demonstrate the frequency shift of neuronal calcium oscillations through infrared stimulation. By deepening our understanding in INS fundamentals, this technique holds great promise for advancing neuroscience, deepening our understanding of pathologies, and potentially paving the way for future clinical applications.

## Introduction

Recent advancements in neurostimulation methodologies have catalyzed breakthroughs in neuroscience and historically have led to novel therapies for neurological disorders. Over the past decades, a spectrum of clinical applications has emerged, such as deep brain stimulation^1^, intraoperative brain mapping^2^, or spinal cord stimulation for chronic pain management^3^, primarily relying on classical electrical stimulation. However, the control of the spatial resolution of electrical stimulation faces challenges due to the conductivity properties of the neural tissue.

To address this issue, optical neurostimulation methods have gathered an increased interest over the last 20 years with optogenetics and infrared neural stimulation (INS), also known as infrared neural modulation (INM) in some recent papers. Both stimulation and inhibition of neural signals can be achieved in this context^4,5^. On one hand, optogenetics offers high spatial selectivity in optimized conditions, as well as cell type specificity through the use of promoters^6,7^. Although it is a powerful tool that has found numerous applications, the need for genetic manipulations makes it less suitable to translate to a clinical context. On the other hand, INS, appears as an alternative of optogenetics in the frame of optical neurostimulation as there is no need of genetic manipulation or any other exogenous agents. Indeed, the wavelength of the light delivered to the tissue is chosen to be close to the absorption peaks of water (including 800–900 nm^8^, 1450–1470 nm^9^, 2120 nm^10^ and 5600 nm^11^, inducing a fast and focal photothermal interaction, which according to mainstream theories is at the origin of the subsequent neural stimulation^12,13^.

Based on this photothermal mechanism, INS has been applied in various targets in both peripheral and central nervous systems, as for instance in the auditory system acutely^14–16^ and chronically^17^, in the vestibular and facial nerves^18–20^, ex vivo on pleural-abdominal connective nerves in the frame of neural inhibition^21^, but also for cardiac pacing^22,23^. In the central nervous system (CNS), INS has demonstrated success in various applications within the cortex. Focal stimulation of the rat somatosensory cortex^24–26^, non-human primate (NHP) visual cortex^27^ and mouse visual cortex^28^ has been achieved. In this frame, coupling INS with electrophysiology recordings, calcium imaging and optical intrinsic imaging revealed that the infrared exposure induces an intensity-dependent neural response and oxygen-related hemodynamic signals. More recently, INS has been integrated with fMRI in cats and NHPs, providing detailed maps of brain connectomes with high specificity for both cortical and subcortical sites^29–31^. These diverse applications have even been extended to the first application in the human cerebral cortex, demonstrating the feasibility of stimulating single cortical nodes within the human CNS using infrared stimulation^12^.

The precise mechanism through which infrared-induced transient heat initiates neural modulation remains unclear. Over the years, multiple hypotheses have been proposed and multiple subsequent mechanisms to infrared exposure were ruled out. In terms of stimulation, the activation of heat sensitive ion channels (TRPV1 and TRPV4) has been demonstrated to be at the origin of initial membrane potential changes leading afterwards to the depolarization through the activation of voltage sensitive ion channels^32,33^. However, depending on the targets and the expression of these channels, TRP channels may not be the primary mechanism of INS in NHP^12^. A more generalized biophysical mechanism has been proposed which relies more on the rapidity of the temperature increase, and associated to plasma membrane capacitance changes, resulting in the generation of capacitive currents explaining the neural stimulation^34,35^. Other investigations have focused on intracellular effects, more specifically around infrared-induced increase of intracellular calcium ion concentration, originating from endoplasmic reticulum stores, via activation of the phospholipase C and the inositol trisphosphate (IP_3_) signaling pathway^36–38^, with potential transfer to mitochondria^39^. In contrast, infrared-induced neural inhibition is believed to arise more from the thermal acceleration of K^+^ channel kinetics, thus blocking neural conduction^24,40^. Additionally, with regard to inhibition, it was also shown that infrared parameters, and more specifically the power threshold could be adjusted according to the length of the axon which is exposed to infrared^41,42^ or through the use of isotonic extracellular ion replacement^43^. In contrast, within the CNS, observed inhibition is more apt to result from the stimulation of inhibitory circuits^12,27^.

While neural networks have been of primary interest in the frame of INS, this technique also has the particularity, in contrast to optogenetics, to be not cell type specific. INS has demonstrated efficacy in stimulating calcium dynamics not only in excitable cells but also in non-excitable cells such as tumor cells^37^, astrocytes^44^ or microglia^45^. This suggests that these effects could be mediated by both neuronal and non-neuronal targets, holding significant potential for the study of astrocytes in neurodegenerative diseases. Indeed, astrocytes functions are diverse and their potential implications in neurodegenerative disorders represent a growing and promising field in neurosciences^46^. However, to the best of our knowledge, there is a lack of studies demonstrating well defined simultaneous astrocytic and neuronal signals in response to INS stimulation.

In this present paper, we demonstrate that trains of short infrared pulses have the capacity to initiate calcium signaling in both astrocytes and neurons from the rostral lumbar spinal cord. First, we demonstrate that a single infrared pulse train is effective in triggering calcium signaling in astrocytes, propagating through the astrocytic network. Second, we demonstrate that multiple infrared pulse trains induces the stimulation of both neurons and astrocytes, each exhibiting distinct temporal and amplitude characteristics. Third, we show that infrared-induced neuronal calcium signaling is triggered by the activity of sodium channels, while infrared-induced astrocytic signaling is strongly dependent on extracellular calcium and TRPV4 channels. Finally, we demonstrate that neuronal calcium oscillations can be frequency-shifted through infrared stimulation.

## Results

### Distinct spatio-temporal profile of calcium signaling in spinal astrocytes and neurons in response to single train of infrared pulses

**Fig. 1:**
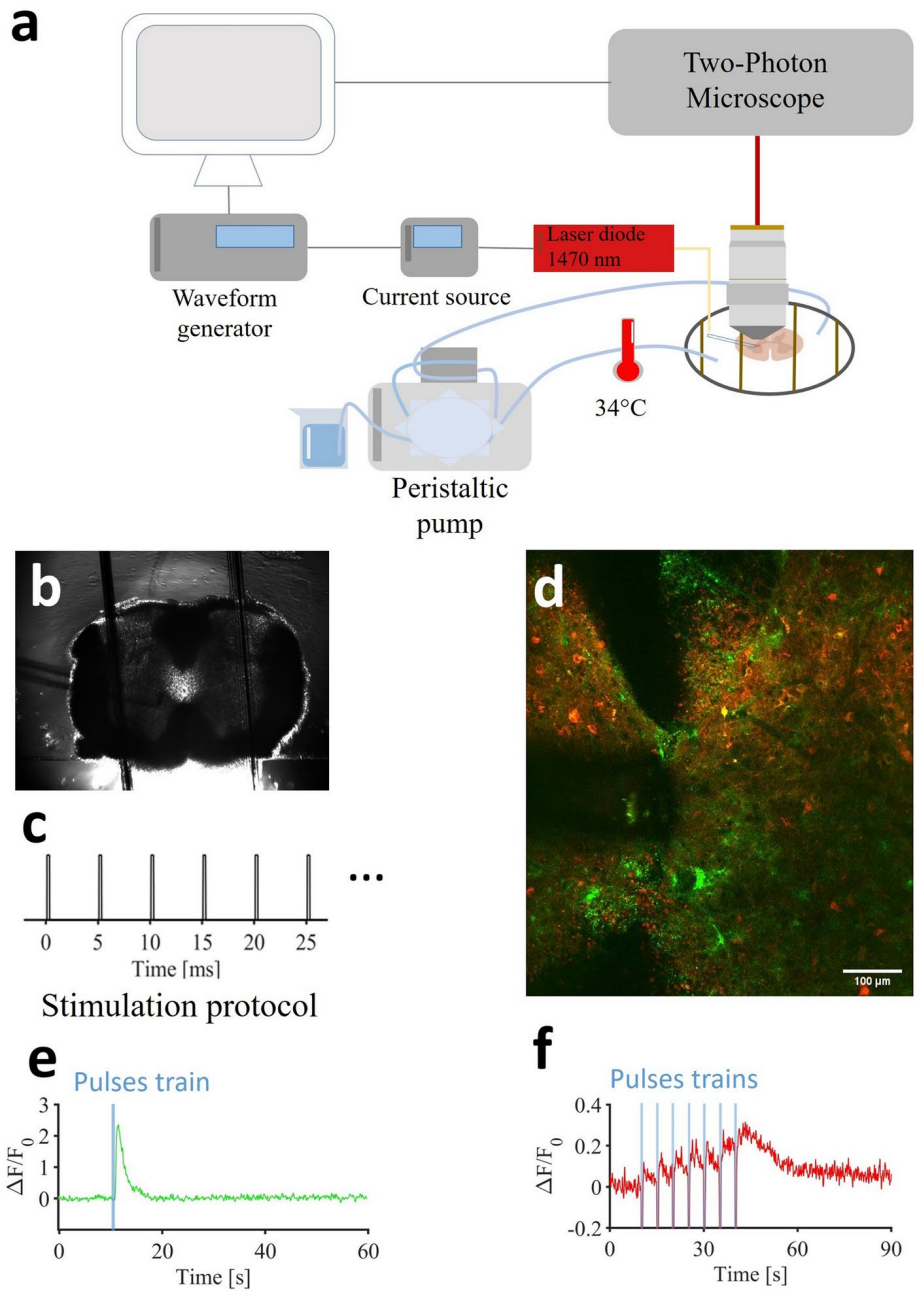
Experimental setup and examples of simultaneous infrared-induced calcium signaling in astrocytes and neurons. (**a**) Experimental setup. (**b**) Wide field image of the spinal cord slice with the optical fiber (arrow) delivering the infrared. (**c**) Infrared stimulus protocol: several pulse trains composed of 250 $\upmu$s pulses repeated at 200 Hz for 500 ms. (**d**) Example of two photon image with GCaMP6f labelled astrocytes (green) and jRGECO1a labelled neurons (red). (**e**) Representative example of infrared-induced calcium signal in astrocytes induced with a single pulse train. (**f**) Representative example of Infrared induced calcium signal in neurons induced with 7 pulse trains.

To investigate the effect of infrared stimulation on neuro-glial responses, we employed a sophisticated experimental setup (Fig. 1a). We performed two-photon calcium imaging of neuronal and astrocyte signals in the rostral part of the lumbar spinal cord (Fig. 1b) known for its powerful rhythmogenic capacity^47^. Mice were injected at birth with AAVs designed to express GCaMP6f in astrocytes (green) and jRGECO1a in neurons (red). In response to infrared stimulation protocols (Fig. 1c), we simultaneously imaged the chromophores in the ventro-medial part of lumbar slices (L1–L2) from post-natal mice (P12–19) (Fig. 1d).The infrared exposure started 10 seconds after the beginning of the recording in order to get a fluorescence baseline. Distinct astrocytic and neuronal calcium signaling were obtained in response to a repetitive pulses of infrared stimulation (Fig. 1e,f).

We initially investigated whether pulsed infrared, delivered in a single train of 100 pulses lasting 250 microseconds at 200 Hz, could elicit intracellular calcium signaling in both astrocytic and neuronal compartments. To investigate this, we applied various trains of pulses with distinct energy densities ranging from 0.40 to 0.66 J cm$^{-2}$. Previous experiments conducted at 0.72 J cm$^{-2}$ showed sustained increase of calcium fluorescence signals suggesting immediate thermal damage and cell toxicity (See supplementary video 1).

**Fig. 2:**
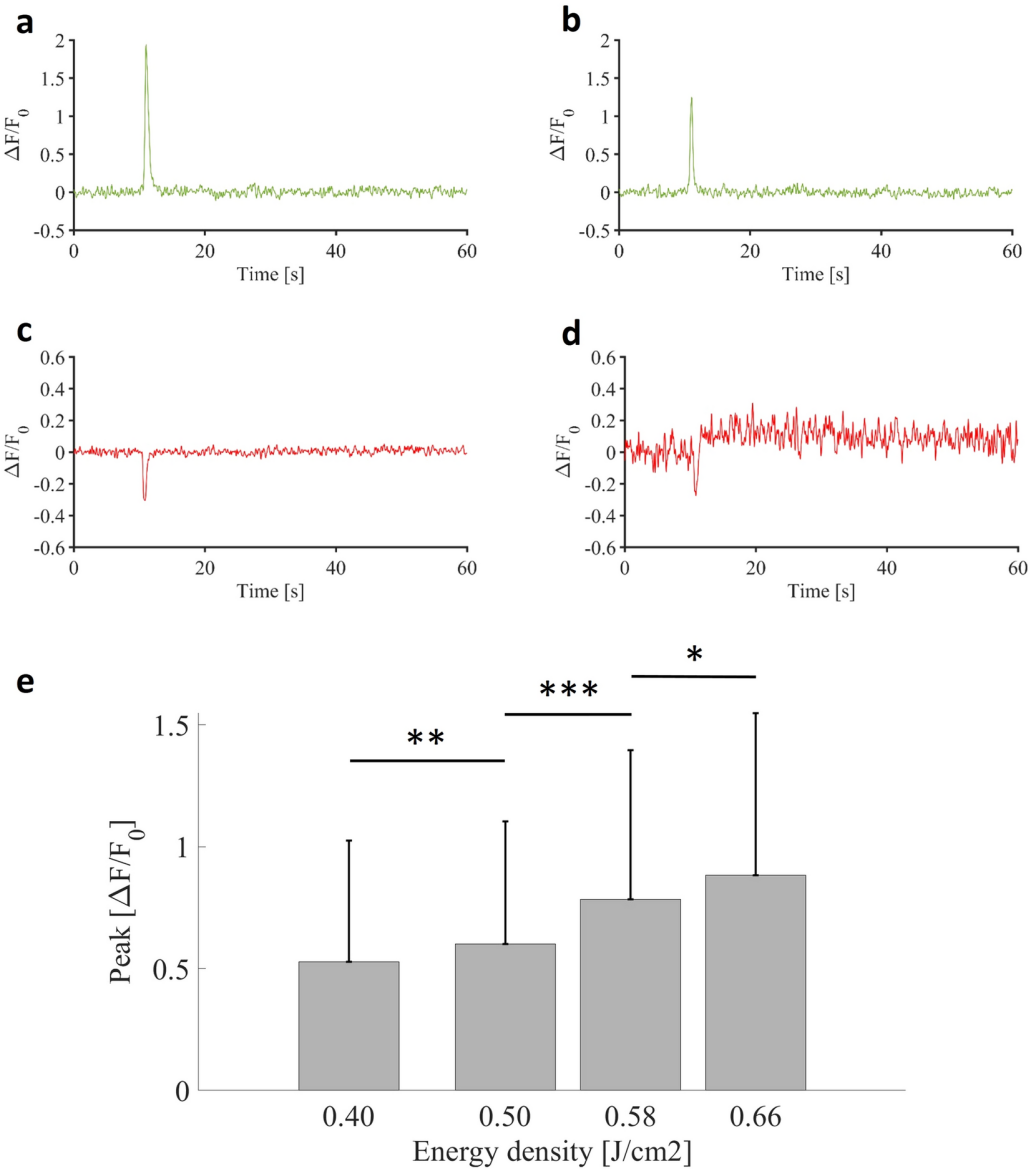
(**a,b**) Examples of normalized astrocytic calcium transients for a single stimulation protocol (250 $\upmu$s pulses repeated at 200 Hz for 500 ms at 0.58 J cm$^{-2}$). (**c**,**d**) Examples of neuronal calcium signaling normalized for a single stimulation protocol (250 $\upmu$s pulses repeated at 200 Hz for 500 ms at 0.58 J cm$^{-2}$). (**e**) Dose-response histogram of infrared induced calcium signaling in astrocytes.

In our case, we obtained physiological responses in astrocytes in a dose-dependent manner at lower intensities (Fig. 2a,b). As a result, the intensity dependency of INS was confirmed with an average response ($$\Delta$$F/$$F_{0})$$ of 0.53 ± 0.49 for 0.40 J cm$$^{-2}$$, 0.60 ± 0.50 for 0.50 J cm$$^{-2}$$, 0.78 ± 0.61 for 0.58 J cm$$^{-2}$$ and 0.88 ± 0.66 for 0.66 J cm$$^{-2}$$ (Fig. 2e). Regarding infrared-induced calcium signaling in neurons, the results were quite different and inhomogeneous. Indeed, the majority of neuronal ROIs did not show any response (Fig. 2c), while in few ROIs we could observe a noisy signal in neurons with a single train of infrared pulses (Fig. 2d),which were hard to describe as a clear response, especially as even in these cases, the reproducibility of infrared-induced neuronal calcium signals was insufficient for performing advanced studies and analyses. Note that these weak signals were primarily detected in neuropil, such as dendrites or axons, rather than in somas. Based on those observations, and considering previous findings in the literature, we hypothetized that we probably triggered some infrared-induced neuronal activity, at the limit of the detection capabilities of calcium imaging with jRGECO1a. In this first part of the present study, we consequently focused on infrared-induced astrocyte calcium signaling. We could observe as well downward deflections, timely correlated with the infrared exposure. Indeed, in addition to INS physiological effects, the temperature rise has auxiliary effects as it can create a thermal lens in water and act on the quantum yield of fluorescent reporters. These results into negative thermal transient deflections in calcium traces^48^. A recent study with INS suggests as well that inhibitory neurons could also bring their contributions to those deflections^49^. However, here, our data don’t allow us to conclude on the exact origin of those deflections.

**Fig. 3:**
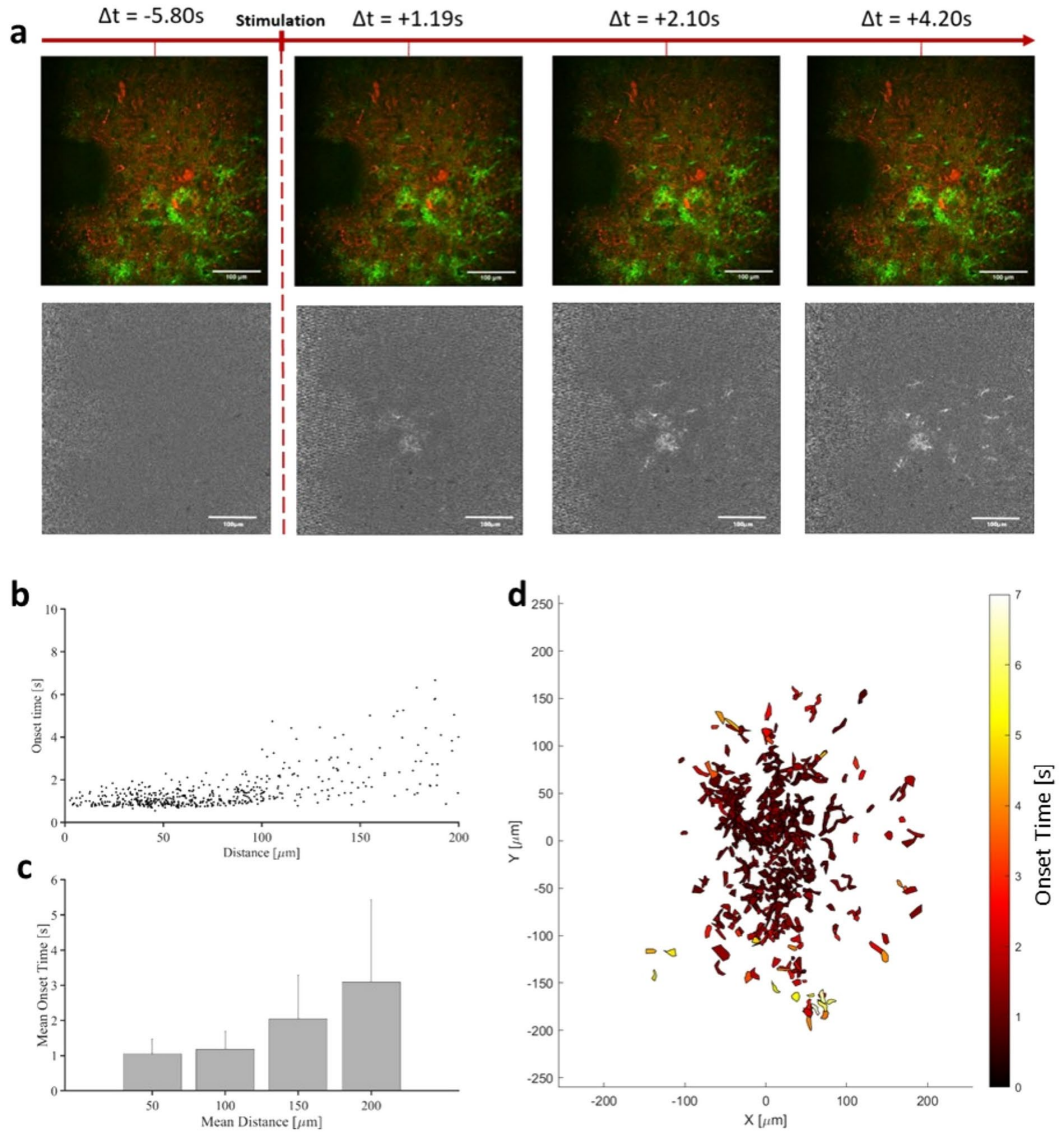
Calcium propagation in astrocytic syncytium for a single stimulation protocol (250 $\upmu$s pulses repeated at 200 Hz for 500 ms at 0.58 J cm$^{-2}$). (**a**) Temporal sequence of raw images (top) and $\Delta$F/F projections (bottom) of infrared induced astrocytic response showing the calcium propagation in the astrocytic syncytium. $\Delta$t values refer to the time compared to the stimulation onset. (**b**) Scatter plot of the onset time for each astrocytic region of interest (represented by a dot) according to the distance to the central part of the beam. (**c**) Average onset time for astrocytic regions of interest located respectively between 0–50 $\upmu$m, 50–100 $\upmu$m, 100–150 $\upmu$m and 150–200 $\upmu$m. (**d**) Heat map of the onset time of the whole dataset centered around the central part of the beam path.

A noteworthy observation was the propagation-like behavior observed in astrocytic calcium signaling triggered by INS, seemingly originating from the center of the beam path (See supplementary video 2). To further investigate and characterize this behavior, we computed the onset time of each single responsive astrocytic ROI. The onset time was defined as the duration between the beginning of the stimulation and the time at which the fluorescence signal passed above the detection threshold. Figure 3b,c shows the onset time of calcium signals in each astrocytic ROI according to the distance to the central part of the beam path. From this representation, two distinct astrocytic populations can be identified. The first population exhibited relatively similar and short onset times, which we may consider as direct stimulation (distance from the center of the beam path below 100 $\upmu$m). The second population was located beyond 100 $\upmu$m, displaying a broader diversity in the onset time, with an average increase with the distance. Moreover, between a radius below 50 $\upmu$m and a radius of 100 $\upmu$m, there was a slight difference between their average onset times. This result might be linked to the Gaussian shape of the beam, creating a short delay between the inner stimulation area and the peripheral one. However, both of these stimulations remain direct. The population of astrocytes exhibiting calcium signaling beyond a distance of 100 $\upmu$m presents later onset times, increasing proportionally with the distance to the center point.

Thus, we were able to calculate the speed of the astrocytic calcium wave propagation, found to be about 52 $\upmu$m/s, similar to calcium waves observed in astrocytic syncytium under physiological conditions.

To provide a comprehensive representation of this astrocytic calcium wave, data from all fields of view (FOVs) and ROIs from various slices and animals were aggregated, superimposed, and centered based on the beam path, as illustrated in Fig. 3d.

### Repeated infrared train of pulses induces astrocytic and neuronal calcium signals

**Fig. 4:**
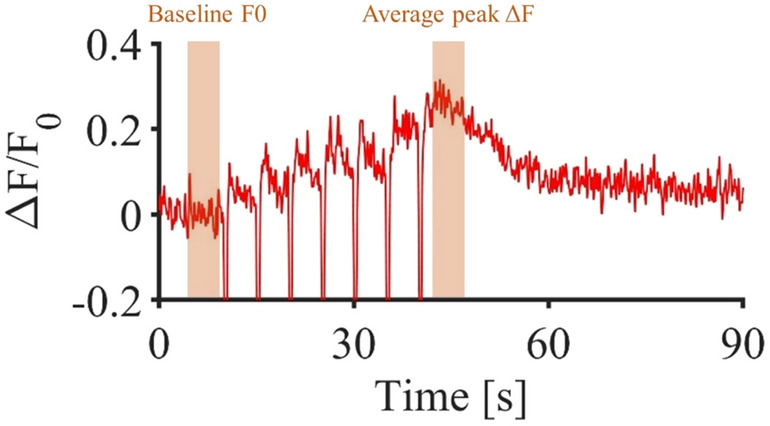
Infrared induced calcium signaling in interneuron soma following 7 pulse trains (250 $\upmu$s pulses repeated at 200 Hz for 500 ms at 0.58 J cm$^{-2}$ repeated each 5 s).

**Fig. 5:**
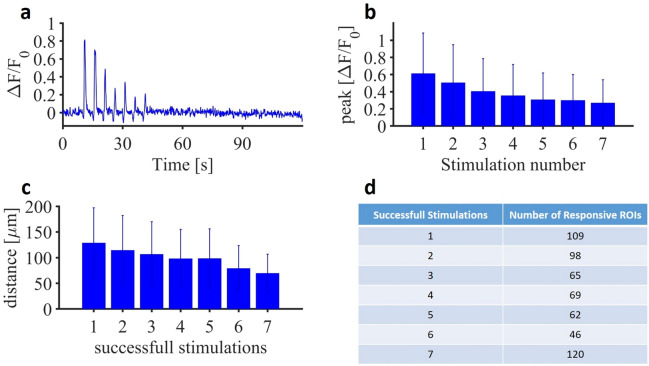
(**a**) Representative example of an astrocyte responding to 7 successive stimulations (250 $\upmu$s pulses repeated at 200 Hz for 500 ms at 0.58 J cm$^{-2}$ repeated each 5 s). (**b**) Evolution of the astrocytic infrared-induced calcium peak amplitude over the successive stimulation for the astrocytes responding to 7 stimulations. (**c**) Average distance of the astrocytic regions of interests according to their respective number of successful stimulations. (**d**) Total of ROIs activated according to their number of successful stimulations out of the 7 successive performed.

To obtain simultaneous neuronal and astrocytic calcium responses, we used repeated pulse train delivered at 0.58 J cm$^{-2}$ with a frequency of 0.2 Hz. This stimulation protocol did not induce any heat accumulation in the tissue that could potentially lead to damage as the time between two infrared pulse train was sufficient for heat to dissipate in the tissue^50^. To better understand the cellular mechanisms underlying spinal rhythmicity, all experiments were conducted in the ventro-medial region of the lumbar spinal cord, where the majority of oscillatory interneurons are located^51^. First, we observed that neuropil exhibited a large panel of calcium signaling responses, either starting after the first pulse train or any of the other ones. Due to this variability, we focused our analysis on calcium signals within the neuronal soma. We observed a cumulative effect of infrared pulses with a slow and progressive increase in the calcium signals in neuronal soma throughout the stimulation period (Fig. 4). This cumulative effect seen in calcium signals was related to a progressive increased firing frequency of the targeted neurons referred as wind-up^47^. Figure 4 shows a representative example of interneuron soma exhibiting signals induced by repeated infrared pulse trains. To quantify the wind-up amplitude, the average signal amplitude was averaged over the 5 seconds following the last pulse train and compared to the average baseline during the 5 seconds preceding the first pulse train. We also observed significant variations of calcium signals from astrocytic ROIs in response to successive stimulations. Contrary to neurons, there was no cumulative increase in intracellular calcium over the stimulation period. Instead, there was a repetition of fast calcium transients with decreasing amplitude over the pulse train (Fig. 5a). Importantly, not all responsive astrocytes exhibited signaling throughout the seven pulse trains; some responded only once, twice, or forth, highlighting a functional diversity in astrocyte signaling. To validate the observed decrease in calcium signals over the pulse trains in a robust manner, we considered only astrocytes responding to all seven pulse trains and extracted the respective values of their calcium peaks (Fig. 5b). In this context, we thus observed a decrease in the infrared-induced calcium signals over the repetitive stimulations, seemingly reaching a plateau after 4 stimulations. Furthermore, considering all the responsive ROIs, we observed that an increased number of successful stimulations correlated positively with a greater proximity of the ROIs to the center of the beam path (Fig. 5c). This finding confirms the spatial selectivity and robustness of stimulation within the beam path of the infrared exposure.

### Distinct sources of infrared-induced calcium signals in spinal neurons and astrocytes

**Fig. 6:**
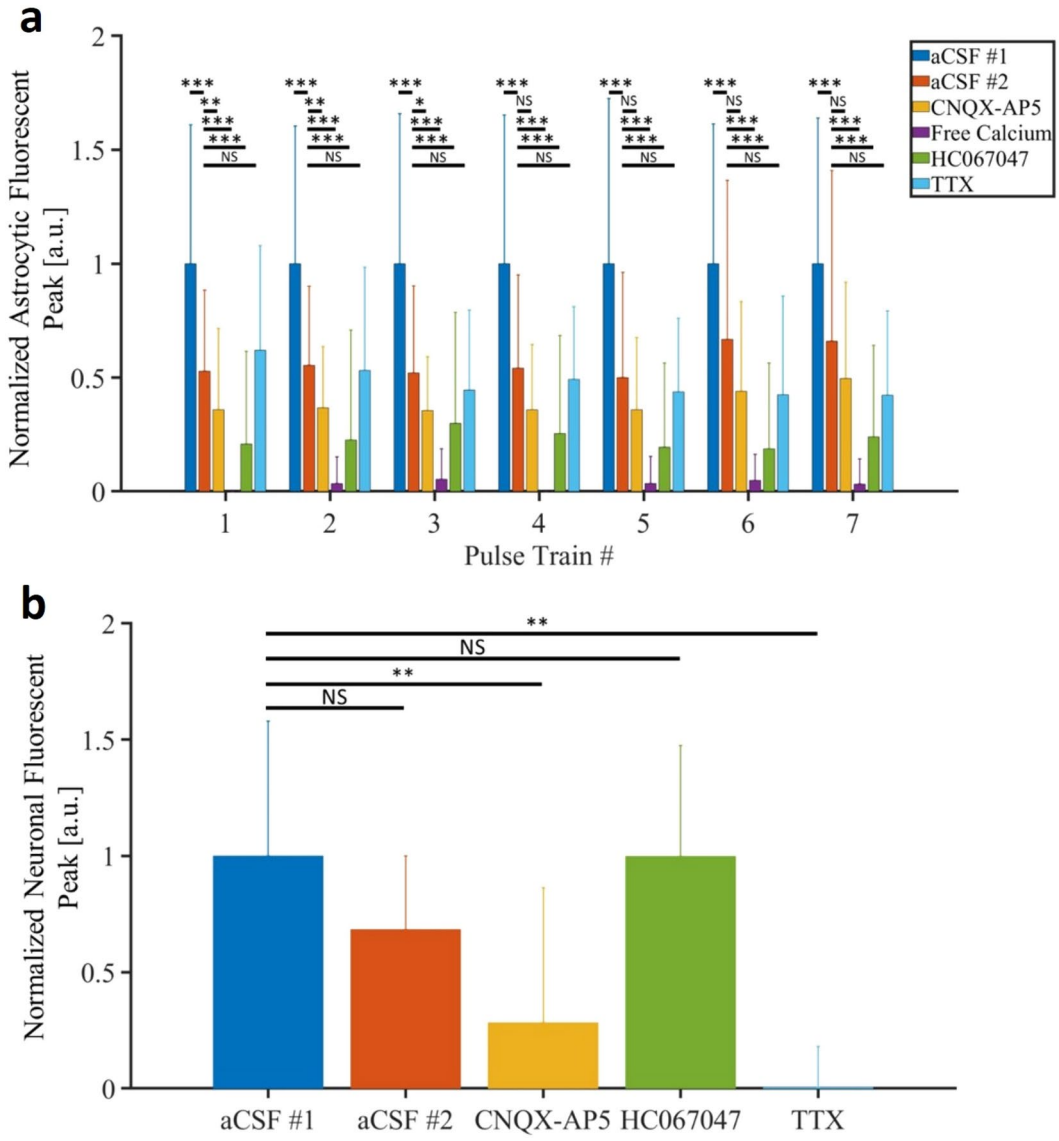
Pharmacological identification of source of calcium in astrocytes (**a**) and neurons (**b**) following 7 infrared pulse trains (250 $\upmu$s pulses repeated at 200 Hz for 500 ms at 0.58 J cm$^{-2}$ repeated each 5 s).

We next investigated the mechanisms underlying the source of repeated infrared-induced calcium signaling in neurons and astrocytes. For that, we conducted investigations using pharmacological inhibitors and ion substitution to understand (i) the origin of calcium signals and (ii) the impact of infrared-induced astrocytic signaling on neuronal signaling and vice versa. We first evaluated the impact of the different drugs modulating the astrocytic or neuronal calcium signaling (Fig. 6). Each drug’s effect was compared with a control experiment conducted prior to drug application. For astrocytes, only ROIs responding to 7 stimulations were considered (Fig. 5d). For neurons, only soma exhibiting wind-up calcium signals were taken into account. The amplitude at the end of the wind-up was compared between the conditions. All results obtained were normalized in amplitude, in order to be able to compare independent sets of data.

We first investigated whether astrocytic and neuronal calcium signaling changed in response to two consecutive infrared stimulation of 10-minutes interval. This allowed us to establish a control value for comparison with the subsequent effects of drugs. The results show a statistically significant reduction in the response induced by INS for astrocytic calcium transients, as indicated by Wilcoxon sign rank p-values ($1.65.10^{-8}$ for stim #1, $7.86.10^{-8}$ for stim #2, $1.27.10^{-6}$ for stim #3, $4.82.10^{-8}$ for stim #4, $3.26.10^{-7}$ for stim #5, $1.27.10^{-4}$ for stim #6, $1.90.10^{-4}$ for stim #7) while the reduction in neuronal wind-ups is not statistically different (Wilcoxon sign rank p-value = 0.13). To eliminate the bias induced by the repetition of stimulations for astrocytes, all subsequent results obtained with different pharmacological drugs has been compared to the data from this second stimulation set using the Mann-Whitney statistical test.

Then, to determine whether the infrared-induced calcium signals were due to calcium entry from the extracellular medium or to calcium release from intracellular stores, we perfused slices with a calcium-free solution^52–54^. In this condition, we showed that astrocytic responses were nearly completely eliminated, pointing towards extracellular calcium as the source of astrocytic calcium signals. However, we cannot rule out that intracellular stores are inactive under normal conditions, though they could likely be triggered by the calcium-induced calcium release process.

For neurons, analysis was precluded due to the use of a free-calcium solution, a condition known to induce neuronal rhythmicity^55^. Indeed, neurons exhibit oscillating properties through the enhancement of persistent sodium currents (INaP) in this specific condition. This phenomenon and impact of infrared exposure in this condition will be discussed in the next section. Given that INS generates highly controlled temperature transients in neural network, we next explored the temperature-sensitive mechanisms responsible for neuroglial calcium dynamics. It is well known that temperature modulates the transient receptor potential vanilloid (TRPV) channels^56,57^. Beyond the TRPV family, we focused on the temperature-sensitive TRPV4 channels because (i) they are expressed in both astrocytes and neurons^58,59^ and (ii) they are activated by non-noxious heat stimuli^60,61^. Thus, spinal slices were perfused with HC067047, a TRPV4 blocker. In this condition, infrared-induced astrocytic calcium signals were significantly reduced compared to control (p < 0.005 for each of the 7 stimulations). However, it is noteworthy that the reduction in calcium signals induced by HC067047 was comparatively less significant than those induced by a low extracellular calcium concentration. This observation suggests that while TRPV4 may contribute, they are not the only source of calcium influx under these conditions. Interestingly, when examining the neuronal responses, application of HC067047 did not modify the wind-up calcium signaling. All together, these observations suggest distinct mechanisms underlying INS for astrocytes and neurons.

We next explored the calcium dynamics in astrocytes and neurons in the presence of tetrodotoxin (TTX) to block voltage-gated sodium channels. Contrary to astrocytes, the infrared-induced calcium signaling in neurons vanished completely under TTX (Fig. 5a,b), indicating that the cumulative increase of calcium in neuronal soma is linked to their increased electrical activity induced by infrared stimulation.

To ascertain the potential involvement of glutamatergic mechanisms in infrared-induced calcium dynamics, we bath applied a cocktail of antagonists targeting the fast glutamatergic synaptic transmission. This cocktail included CNQX and DL-AP5 blocking AMPA and NMDA receptors, respectively. Interestingly we observed significant effects of CNQX/DL-AP5 on both infrared-induced astrocytic and neuronal calcium signals. On the one hand, a statistically significant decrease in the amplitude of infrared-induced activity in astrocytes was noted for the first three stimulation train, whereas it was non-significant for the four latest ones. On the other hand, the neuronal calcium wind-up was also statistically significantly smaller with application of CNQX-AP5 compared to control experiments. Altogether, these results suggest that glutamate plays a role in both infrared-induced astrocytic and neuronal calcium signals.

### Infrared-induced modulation of neuronal oscillations

To further explore the capacity of infrared stimulation to modulate neuronal rhythmicity, we used a calcium-free solution. As mentioned earlier, in this condition, most neuronal somas exhibited spontaneous bursting properties in hippocampus^62^ or spinal cord^50,63^. The main hypothesis behind this phenomenon suggests that lowering the extracellular calcium concentration induces a shift in INaP activation threshold towards more negative potentials, enhancing their amplitudes and inducing a pacemaker-like behavior of the neurons. This INaP-dependent bursting property made the pacemaker network hypothesis a valuable model to account for locomotor rhythm generation over the years^55^.

**Fig. 7:**
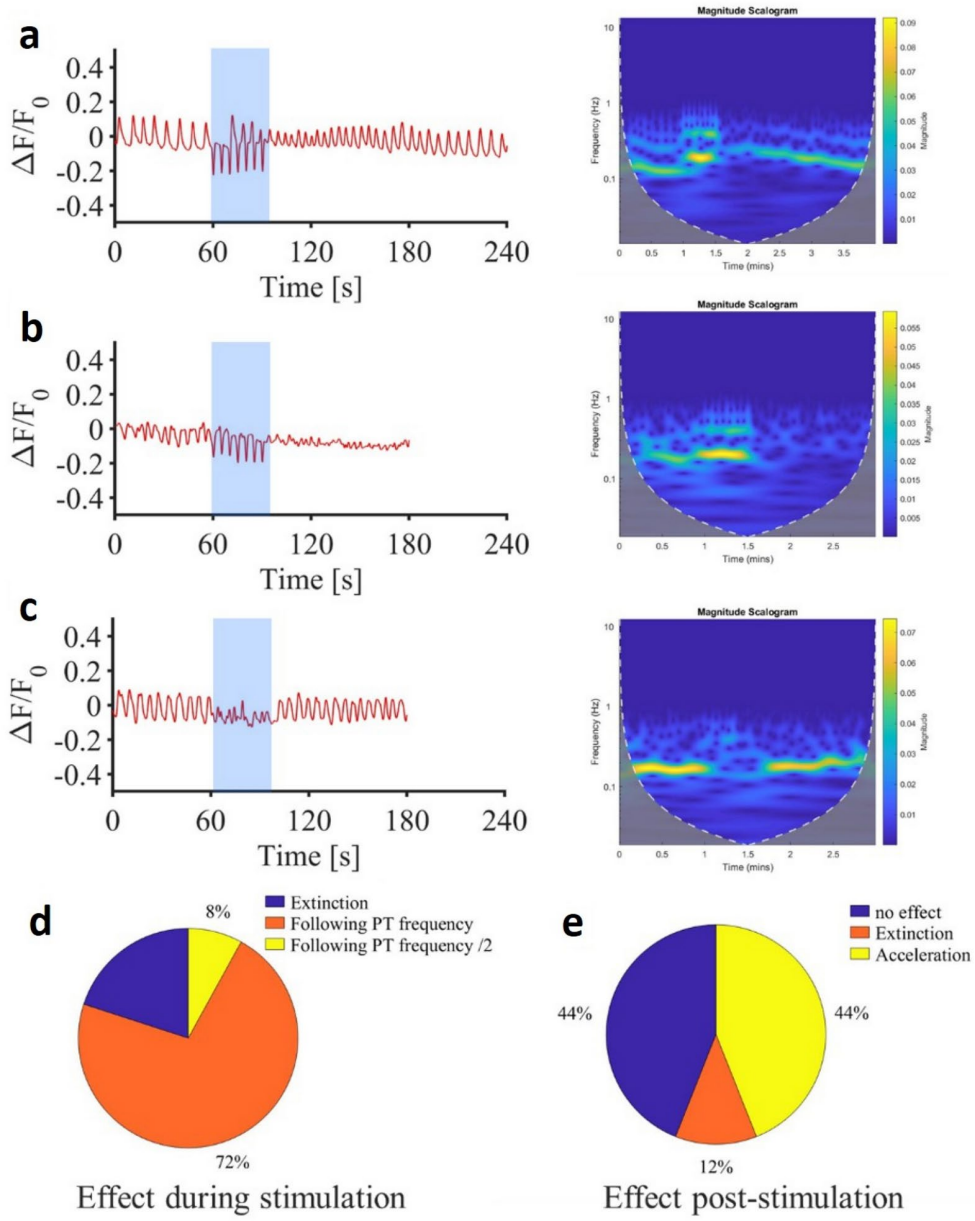
Time courses and time frequency analysis of interneuron bursting frequency alteration induced by infrared stimulation (7 infrared pulse trains of 250 $\upmu$s pulses repeated at 200 Hz for 500 ms at 0.58 J cm$^{-2}$ repeated each 5 s). (**a**) Example of frequency acceleration post stimulation. (**b**) Example of frequency extinction post stimulation. (**c**) Example of absence of effect post stimulation. (**d**) Pie chart sorting pro-stimulation effects (*PT* pulse trains). (**e**) Pie chart sorting post-stimulation effects.

Here, using the same infrared multiple trains stimulation, we show that infrared can modulate the bursting frequency in many different ways: among over 25 bursting neurons with a spontaneous bursting frequency between 0.1 and 0.18 Hz (in 8 different field of views of 3 slices taken from 3 different animals) exposed to our infrared protocols (Fig. 7d), 5 exhibited bursting activity extinction during the infrared protocol, 18 followed the infrared pulse train frequency (0.2 Hz) and 2 responded to 1 pulse train out of 2 (frequency = 0.1 Hz). Interestingly, transient changes in bursting frequencies were also observed post-stimulation (Fig. 7e). Among the same 25 neurons, 11 got a sustained increase bursting frequency, gradually returning to the initial spontaneous value progressively over 90–120 s (Fig. 7a), 11 returned to the initial spontaneous bursting activity immediately after the stimulation (Fig. 7c), and 3 showed termination of their bursting activity (Fig. 7b).

## Discussion

This study presents compelling evidence of infrared-induced modulation of both astrocytic and neuronal calcium signaling within the spinal locomotor networks. We first reported a distinct response pattern in astrocytes compared to neurons. Contrary to neurons, astrocytes exhibited a robust transient increase in calcium following a single train of infrared pulses, characterized by a centrifugal propagation-like spread along the beam path. Interestingly, while astrocytes calcium responses diminished in amplitude over successive pulses, neuronal responses exhibited a sustained increase. Indeed, ventromedial neurons displayed a cumulative increase of calcium fluorescence signals, known as windup phenomenon, in response to a repetitive train of infrared pulses. Further investigation revealed key differences in the underlying mechanisms. We demonstrated that infrared-induced astrocytic calcium signals are (i) TTX-insensitive, (ii) mainly dependent on extracellular calcium and (iii) strongly mediated by the temperature-sensitive TRPV4 channels. In contrast, neuronal calcium signaling was (i) TTX-sensitive, (ii) modulated by the fast glutamatergic synaptic transmission and (iii) TRPV4-insensitive. Finally, our findings demonstrated that infrared pulses have the capacity to modulate the rhythmicity of oscillatory ventromedial spinal neurons. To date, only one study has hinted at the concurrent modulation of astrocytic and neuronal responses through infrared illumination^26^. This particular in vivo investigation was conducted in the rat somatosensory cortex, employing the same single infrared pulse train utilized in our study. Using Oregon Green 488 BAPTA-1AM as a calcium dye, the authors monitored calcium dynamics in both neurons and astrocytes. They distinguished fast and slow components of the infrared-induced calcium signals, attributing them to neurons and astrocytes, respectively. Their findings suggested that astrocytes may primarily drive the calcium signals evoked by INS, with neurons showing less pronounced activation. Although direct neuronal activation was not observed, they did detect a neuropil response, potentially involving apical dendrites, axons, and astrocytic processes.

In our study, we aimed to decipher the cellular mechanisms underlying the infrared-induced calcium dynamics in spinal astrocytes and neurons. We demonstrated that a single infrared pulse train (100 pulses of 250 $\upmu$s at 200Hz) elicited calcium signaling in astrocytes with evidence of activation of the astrocytic network through the propagation of the activity in nearby areas not exposed to infrared as indicated by the evolution of the onset time. This induced activity was dose dependent as increasing the infrared energy density deposited resulted in an increased amplitude of the induced calcium signals. The damage threshold was found to be at 0.72 J cm$^{-2}$, indicated by a sustained increase of the fluorescence signal in cells and neuropil, suggesting immediate thermal damage and cell toxicity^62^. This damage threshold aligns with the thresholds previously found in literature in the CNS of rats, NHPs and humans^12,50^. However, the single infrared pulse train did not elicit a strong and reproducible neuronal activity. With the single train of infrared pulses, we could indeed observe a few calcium signals in neurons, primarily in neuronal projections, i.e. dendrites or axons, rather than in somas. This suggests that the neuropil response observed by Cayce and colleagues had a contribution from neuronal projections, Nevertheless, the reproducibility of infrared-evoked neuronal calcium signals was not robust enough to perform advanced analyses. Our main hypothesis is that the calcium sensor used here (jRGECO1a) may be not sensitive enough to report weak changes in neuronal activities in two photon imaging, as it was shown in previous work in mouse visual cortex that calcium transients elicited by the same infrared paradigm was very low as reported by GCaMP6f^28^. While future work may explore combining this approach with electrophysiology to confirm the hypothesis, in this study, we chose to repeat the infrared pulse trains to obtain more robust neuronal responses.

Consequently, we applied a repetitive infrared pulse train to induce calcium signals in both jRGECO1a labelled neurons and GCaMP6f labeled astrocytes with different temporal and amplitude profiles.

Regarding the astrocytic signaling, the different pulse trains elicited repeated robust transients calcium signals similar to those observed with a single pulse train. However, not all astrocytic ROIs exhibited calcium transients for each infrared pulse train. Over the course of 7 pulse trains, only a subset of astrocytes showed activity all 7 times, while others responded between 1 and 6 times. By sorting the astrocytic ROIs according to the successful stimulations, we observed that the higher the number of successful stimulations, the closer the ROIs were from the center of the beam path. This result confirms the spatial selectivity and robustness of stimulation in the beam path of the infrared exposure. We next focused on the astrocytic population responding to all 7 pulse trains. Notably, there was a decrease in the infrared-induced calcium signals over repetitive stimulations, reaching a plateau after 4 stimulations. This aligns with the fact that internal calcium stores may play a role in the elicited calcium signals, and repetitive stimulations might deplete these stores, which do not have sufficient time to refill, ultimately reaching a plateau once the stores are completely empty^63^. Yet the calcium signals do not vanish completely, suggesting as well a first contribution from extracellular calcium which enters into the cell triggering a calcium-induced calcium release phenomenon. To investigate on the origin of the calcium transient in astrocytes, we applied low extracellular calcium concentration which completely inhibited the elicited transients while HC067047, a specific TRPV4 antagonist, only reduced them. These results indicate that extracellular calcium is essential for infrared induced astrocytic activity in the spinal cord, while the cationic TRPV4 channels are minor contributors to the astrocytic calcium response. Application of TTX had no effect, suggesting that infrared-induced astrocytic activity is independent of neuronal sodium activity. Interestingly the use of the cocktail of glutamate ionotropic receptor antagonists (CNQX-AP5), induced a statistically significant decrease only in the first three pulse train stimulations.

In neurons, the different pulse trains elicited a gradual intracellular calcium increase over the total duration of the pulse trains. This progressive increase is referred as wind-up phenomenon and is usually defined as a progressive and frequency-dependent facilitation of the responses of a spinal cord neuron which can be observed upon the application of constant and repetitive stimuli^64^. Application of TTX completely inhibited the wind-ups induced by infrared, while HC067047 had no effect. Alltogether, these observations highlight two different mechanisms underlying the neuronal and astrocytic calcium signals. Thus, they are likely correlated with an infrared-induced increase in spike frequency. Furthermore, the blockade of NMDA and AMPA receptors significantly reduced the amplitude of wind-ups aligning with the involvement of glutamatergic receptors in the generation mechanism of wind-ups. The prevailing theory is that wind-up is mediated by the activation of NMDA receptors, recruited through the relief of magnesium block induced by the cumulative depolarization occurring in the frame of repetitive stimulation^65,66^. NMDA antagonists have been shown in the past to block wind-up generation in various targets as WDR neurons^67^, ventral horn neurons^68^ and motoneurones^69^. As far as physiological relevance, wind-up is known to contribute to the sensory experience of temporal summation, i.e. the increase in the perception of pain in response to repeated or prolonged application of noxious stimuli^65,70^. In this paper, we investigated separately the effect of drugs on neurons and astrocytes responses to INS. Although, all of these results pave the way as well to the fact that it is possible that INS effect might be based on a network mechanism. Further studies should be conducted in this way.

Finally, to explore the influence of infrared neural stimulation (INS) on spinal rhythmicity, we recorded neuronal oscillations in calcium free solution. Across the full stimulation duration (30 seconds) and post-stimulation period, we observed diverse effects on neuronal oscillatory patterns. In some instances (n = 5/25), the infrared protocol suppressed the bursting pattern of ventromedial interneurons, while in others (n = 18/25), these interneurons synchronized with the frequency of the infrared pulse train (0.2 Hz). We also noted transient changes in bursting frequencies post-stimulation, either as an increase lasting 90-120 seconds (n = 11/25) or as an abolishment of bursting activity (n = 3/25). Some also showed no alteration in the oscillatory pattern (n = 11/25). The broad range of effects on neuronal rhythmicity induced by infrared stimulation may stem from the varied subtypes of oscillatory neurons within the spinal locomotor CPG^51^. The oscillatory behavior of spinal interneurons is heavily influenced by glutamatergic synaptic transmission^71,72^, which is engaged by repetitive infrared pulses. Further investigation into these observations is warranted to elucidate the extent and manner in which each neuronal subtype within the locomotor CPG is modulated by infrared stimulation.

Altogether, it should also be considered that this full study was done in an *ex-vivo* context, with spinal cord slices preparation. This model allowed us a large flexibility in the experiment design, but future work will focus on the confirmation of all of the observed effects in an in vivo environment.

Given the critical role of glutamatergic ventromedial neurons in restoring walking post-spinal cord injury, our findings on infrared modulation of spinal rhythmicity offer potential for innovative therapeutic strategies in functional rehabilitation. Moreover, the ability to manipulate neuronal bursting frequency holds implications beyond the spinal cord, offering insights into understanding and treating CNS neurodegenerative diseases. For instance, harnessing modulation of the beta-band neural oscillations to enhance therapeutic interventions in the frame of Parkinson’s disease^73,74^, or targeting gamma-band neural oscillations in Alzheimer’s disease could emerge as promising applications for INS in the future^75–77^.

In conclusion, gaining a deeper understanding of the INS impact on bursting activities across various CNS areas holds significant promise for advancing neuroscience, deepening our understanding of pathologies, and potentially paving the way for future clinical applications. Compared to electrical stimulation, INS presents advantages in terms of spatial precision and eliminates the need for genetic modifications or the use of exogenous chromophores. Despite the absence of standardized equipment for INS, ongoing developments are rapidly emerging, opening up new possibilities^78–81^, including the recent validation of INS for chronic deep brain stimulation (DBS)^82^. However, the path to clinical applications will require advancements both in fundamental understanding of INS mechanisms and in technological innovations to fully exploit the potential of this neurostimulation method.

## Methods

### Mice and ethical standards

CD-1 mice were obtained from Charles River (strain code 022). Mice of both sexes were used in all experiments. Animals were housed on a 12 hr day/night cycle with ad libitum access to water and food. The room temperature was maintained around 22 $^{\circ }$C.

All animal care and use conformed to French regulations (Décret 2010-118) and were approved by the local ethics committee (Comité d’Ethique en Neurosciences INT-Marseille, CE71 Nb A1301404, authorization Nb APAFIS #2018110819197361).

The authors complied with the ARRIVE guidelines.

### Intrathecal vector delivery

A minimally-invasive technique was used to micro-inject adeno-associated viral (AAV) vectors into the T13-L1 intervertebral space. Briefly, in pups cryoanesthetized at birth, the intervertebral space was widened by flexing the spine slightly. The tip of the microcapillary preloaded with the AAV particles was lowered into the center of the T13-L1 intervertebral space. pZac2.1-gfaABC1D-cyto-GCaMP6f (Addgene #52925-AAV5) and pAAV.Syn.NES-jRGECO1a.WPRE.SV40 (Addgene #100854-AAV9) were simultaneously injected and labelled astrocytes and neurons, respectively. A total volume of 2 $\upmu$L /animal was progressively injected (1 $\upmu$L/5s).

### Slice preparation

Mice (post-natal days 12–19, P12-P19) were anaesthetized with intraperitoneal injection of a mixture of ketamine/xylazine (100mg/kg and 10 mg/kg, respectively). They were then decapitated, eviscerated and the spinal cord removed by laminectomy, and placed in a Sylgard-lined petri dish with ice cold (1–2 $^{\circ }$C) aCSF containing (in mM): 252 sucrose, 3 KCl, 1.25 $\hbox {KH2PO}_{4}$, 4 $\hbox {MgSO}_{4}$, 0.2 $\hbox {CaCl}_{2}$, 26 $\hbox {NaHCO}_{3}$, 25 D-glucose, pH 7.4, bubbled with 95% $\hbox {O}_{2}$ and 5% $\hbox {CO}_{2}$. The meninges were removed and the spinal cord (T13-L6) imbedded in a 4% agarose solution. For slicing, an ice-cold solution (1–2 $^{\circ }$C) was used containing (in mM): 130 K-gluconate, 15 KCl, 0.05 EGTA, 20 HEPES, 25 D-glucose, 3 kynurenic acid, and pH 7.4 with NaOH. Spinal slices (325 $\upmu$m) were prepared from the L1–L2 region and were immediately transferred into the holding chamber filled with bubbled (95% $\hbox {O}_{2}$ and 5% $\hbox {CO}_{2}$) recording aCSF composed of (in mM): NaCl (120), KCl (3), $\hbox {NaH2PO}_{4}$ (1.25), $\hbox {MgSO}_{4}$ (1.3), $\hbox {CaCl}_{2}$ (1.2), $\hbox {NaHCO}_{3}$ (25), D-glucose (20), pH 7.4, 30–32 $^{\circ }$C. After a 30–60 min resting period, individual slices were transferred to a recording chamber continuously perfused (3ml/min) with recording aCSF heated to 32–35 $^{\circ }$C.

### Two-photon Ca2+ imaging

Ex-vivo calcium imaging was performed with a dual-scan two-photon microscope (FemtoS-Dual, Femtonics Ltd, Budapest, Hungary) and a 20X immersion objective (Olympus XLUMPlanFL N20x, 1.00, Olympus America, Melville, NY). The wavelength was tuned at 955 nm to optimize the signal-noise ratio for both calcium sensors, GCaMP6f (astrocytes) and jRGECO1a (neurons). The whole system was controlled through MESc software (Femtonics Ltd, Budapest, Hungary). All recordings were performed in resonant scanning mode at  30 Hz on a single acquisition plane.

### Infrared stimulation

The stimulation was performed with a 1470 nm multimodal diode (LU1470T015, Lumics) mounted on the appropriate cooling block (LU_CB_T_0, Lumics) and coupled to a 105 um diameter optical fiber (numerical aperture of 0.15). The fiber was mounted in a homemade cannula, to bring the tip of it to the surface of the slice with a micromanipulator (LN Junior LR, Luigs-Neumann, Ratingen, Germany). The mounted optical fiber was positionned over the top of the spinal cord slice in the field of view thanks to IR camera guidance. The laser diode was controlled by a current controller unit (LU_DR_AD, Lumics) to set intensity and temporal parameters of the light pulse trains. This unit was externally controlled by a waveform generator (3390 waveform generator, Keithley) and connected to the MESc acquisition software, triggering infrared exposure 10 seconds after the beginning of the recording. The stimulation consist in a repetition of train of 100 pulses of 250 us at 200 Hz for a total time of 500 ms. In the multiple stimulation protocol, 7 similar trains were repeated at a frequency of 0.2 Hz for a total time of 30 seconds of stimulation. The average power delivered infrared exposure was measured using a S145C integrating sphere photodiode power sensor attached to the PM100D power meter (Thorlabs).

### Pharmacology

All solutions were oxygenated with 95% $\hbox {O}_{2}$/5% $\hbox {CO}_{2}$. $\hbox {Ca}^{2+}$-free solution was made by removing $\hbox {Ca}^{2+}$- chloride from the recording aCSF and replacing it with an equimolar concentration of magnesium chloride. All salt compounds and drugs were dissolved in water and added to the recording aCSF. Only HC067047 were dissolved in DMSO (0.1%) and then diluted in recording aCSF. 6-Cyano-7-nitroquinoxaline-2,3-dione (CNQX, #5.04914), D-(-)-2-Amino-5-phosphonopentanoic Acid (DL-AP5; #165304), were obtained from Sigma-Aldrich. Tetrodotoxin (TTX; #1078) and HC067047 (#4100), was obtained from Tocris Bioscience. Kynurenic Acid (#HB0363) was obtained from HelloBio.

### Data analysis

The fluorescence signals were processed in a first time with MESc software (Femtonics Ltd, Budapest, Hungary). Regions of interest (ROI) were manually selected and both raw calcium fluorescence traces and coordinates of the ROIs were exported. Raw fluorescence signals, correlations with coordinates and statistical analyses were all performed with custom Matlab algorithms. Raw fluorescence traces were filtered with a Gaussian filter of 10 points and calcium transients were automatically detected according to threshold detection with a threshold of 4 times the standard deviation of the baseline.

### Statistics

As far as statistical analysis, no statistical method was used to predetermine sample size and group measurements were expressed as mean ± SEM. When two independent groups were compared, Mann-Whitney test was used to assess statistical difference significance. When two conditions (control vs drugs) were compared into the same groups of cells, we used the Wilcoxon matched pairs test was used to assess statistical difference significance.

## Supplementary Information

Supplementary Video 1.

Supplementary Video 2.

Supplementary Information 3.

## Data Availability

All data and codes used and generated during this study will be shared by the corresponding author (David Moreau, david.moreau@emse.fr) upon request.

## References

[1] Breit, S., Schulz, J. B. & Benabid, A.-L. Deep brain stimulation. *Cell Tissue Res.*** 318**, 275–288 (2004). 15322914 10.1007/s00441-004-0936-0

[2] Yamao, Y. *et al.* Intraoperative brain mapping by cortico-cortical evoked potential. *Front. Hum. Neurosci.*** 15**, 635453 (2021). 33679353 10.3389/fnhum.2021.635453 PMC7930065

[3] Barolat, G. Spinal cord stimulation for chronic pain management. *Arch. Med. Res.*** 31**, 258–262 (2000). 11036175 10.1016/s0188-4409(00)00075-8

[4] Zhu, X., Lin, J.-W. & Sander, M. Y. Bidirectional modulation of evoked synaptic transmission by pulsed infrared light. *Sci. Rep.*** 12**, 14196 (2022). 35987765 10.1038/s41598-022-18139-2 PMC9392733

[5] Zhu, X., Lin, J.-W., Turnali, A. & Sander, M. Y. Single infrared light pulses induce excitatory and inhibitory neuromodulation. *Biomed. Opt. Express ***13**, 374–388 (2022). 35154878 10.1364/BOE.444577 PMC8803021

[6] Emiliani, V. *et al.* Optogenetics for light control of biological systems. *Nat. Rev. Methods Primers*** 2**, 55 (2022). 37933248 10.1038/s43586-022-00136-4 PMC10627578

[7] Shemesh, O. A. *et al.* Temporally precise single-cell-resolution optogenetics. *Nat. Neurosci. ***20**, 1796–1806 (2017). 29184208 10.1038/s41593-017-0018-8 PMC5726564

[8] Jiang, B. *et al.* Inhibitory effect of 980-nm laser on neural activity of the rat’s cochlear nucleus. *Neurophotonics*** 6**, 035009–035009 (2019). 31482103 10.1117/1.NPh.6.3.035009 PMC6710856

[9] Throckmorton, G. *et al.* Identifying optimal parameters for infrared neural stimulation in the peripheral nervous system. *Neurophotonics ***8**, 015012–015012 (2021). 33816649 10.1117/1.NPh.8.1.015012 PMC8010905

[10] Cayce, J. M. *et al.* Infrared neural stimulation of human spinal nerve roots in vivo. *Neurophotonics*** 2**, 015007–015007 (2015). 26157986 10.1117/1.NPh.2.1.015007 PMC4478764

[11] Liu, X. *et al.* Nonthermal and reversible control of neuronal signaling and behavior by midinfrared stimulation. *Proc. Natl. Acad. Sci.*** 118**, e2015685118 (2021). 33649213 10.1073/pnas.2015685118 PMC7958416

[12] Ping, A. *et al.* Targeted optical neural stimulation: a new era for personalized medicine. *Neuroscientist*** 29**, 202–220 (2023). 34865559 10.1177/10738584211057047

[13] Wells, J. *et al.* Biophysical mechanisms of transient optical stimulation of peripheral nerve. *Biophys. J. ***93**, 2567–2580 (2007). 17526565 10.1529/biophysj.107.104786 PMC1965456

[14] Richter, C.-P. *et al.* Optical stimulation of auditory neurons: effects of acute and chronic deafening. *Hear. Res.*** 242**, 42–51 (2008). 18321670 10.1016/j.heares.2008.01.011 PMC3431617

[15] Rajguru, S. M. *et al.* Optical cochlear implants: Evaluation of surgical approach and laser parameters in cats. *Hear. Res. ***269**, 102–111 (2010). 20603207 10.1016/j.heares.2010.06.021 PMC2937260

[16] Izzo, A. D., Richter, C.-P., Jansen, E. D. & Walsh, J. T. Jr. Laser stimulation of the auditory nerve. *Lasers Surg. Med. ***38**, 745–753 (2006). 16871623 10.1002/lsm.20358

[17] Matic, A. I. *et al.* Behavioral and electrophysiological responses evoked by chronic infrared neural stimulation of the cochlea. *PLoS ONE*** 8**, e58189 (2013). 23505466 10.1371/journal.pone.0058189 PMC3591411

[18] Bec, J.-M. *et al.* Characteristics of laser stimulation by near infrared pulses of retinal and vestibular primary neurons. *Lasers Surg. Med.*** 44**, 736–745 (2012). 23018648 10.1002/lsm.22078

[19] Teudt, I. U., Nevel, A. E., Izzo, A. D., Walsh, J. T. Jr. & Richter, C.-P. Optical stimulation of the facial nerve: a new monitoring technique?. *Laryngoscope*** 117**, 1641–1647 (2007). 17607145 10.1097/MLG.0b013e318074ec00 PMC3471076

[20] Rajguru, S. M., Rabbitt, R. D., Matic, A. I., Highstein, S. M. & Richter, C.-P. Selective activation of vestibular hair cells by infrared light. *Biophys. J. ***98**, 507a (2010).

[21] Zhuo, J. *et al.* Selective infrared neural inhibition can be reproduced by resistive heating. *Neuromodul. Technol. Neural Interface*** 26**, 1757–1771 (2023). 10.1016/j.neurom.2022.12.004 PMC10366334 36707292

[22] Ford, S., Watanabe, M. & Jenkins, M. A review of optical pacing with infrared light. *J. Neural Eng. ***15**, 011001 (2017). 10.1088/1741-2552/aa795f PMC6002813 28612757

[23] Jenkins, M. W. *et al.* Optical pacing of the adult rabbit heart. *Biomed. Opt. Express*** 4**, 1626–1635 (2013). 24049683 10.1364/BOE.4.001626 PMC3771833

[24] Ganguly, M. *et al.* Voltage-gated potassium channels are critical for infrared inhibition of action potentials: an experimental study. *Neurophotonics*** 6**, 040501–040501 (2019). 31620544 10.1117/1.NPh.6.4.040501 PMC6792434

[25] Cayce, J. M., Friedman, R. M., Jansen, E. D., Mahavaden-Jansen, A. & Roe, A. W. Pulsed infrared light alters neural activity in rat somatosensory cortex in vivo. *Neuroimage ***57**, 155–166 (2011). 21513806 10.1016/j.neuroimage.2011.03.084 PMC3108823

[26] Cayce, J. M. *et al.* Calcium imaging of infrared-stimulated activity in rodent brain. *Cell Calcium*** 55**, 183–190 (2014). 24674600 10.1016/j.ceca.2014.01.004 PMC4014070

[27] Cayce, J. M. *et al.* Infrared neural stimulation of primary visual cortex in non-human primates. *Neuroimage ***84**, 181–190 (2014). 23994125 10.1016/j.neuroimage.2013.08.040 PMC4120263

[28] Kaszas, A. *et al.* Two-photon gcamp6f imaging of infrared neural stimulation evoked calcium signals in mouse cortical neurons in vivo. *Sci. Rep. ***11**, 9775 (2021). 33963220 10.1038/s41598-021-89163-x PMC8105372

[29] Yao, S. *et al.* Functional topography of pulvinar-visual cortex networks in macaques revealed by ins-fmri. *J. Compar. Neurol.*** 531**, 681–700 (2023). 10.1002/cne.25456 36740976

[30] Xu, A. G. et al. Focal infrared neural stimulation with high-field functional mri: a rapid way to map mesoscale brain connectomes. *Sci. Adv.*** 5**, eaau7046 (2019). 10.1126/sciadv.aau7046 PMC6482007 31032400

[31] Shi, S. *et al.* Infrared neural stimulation with 7t fmri: A rapid in vivo method for mapping cortical connections of primate amygdala. *Neuroimage ***231**, 117818 (2021). 33548458 10.1016/j.neuroimage.2021.117818 PMC9947864

[32] Suh, E., Matic, A. I., Otting, M., Walsh Jr, J. T. & Richter, C.-P. Optical stimulation in mice lacking the trpv1 channel. In *Photons and neurons*, vol. 7180, 112–116 (SPIE, 2009).

[33] Albert, E. *et al.* Trpv4 channels mediate the infrared laser-evoked response in sensory neurons. *J. Neurophysiol. ***107**, 3227–3234 (2012). 22442563 10.1152/jn.00424.2011

[34] Plaksin, M., Shapira, E., Kimmel, E. & Shoham, S. Thermal transients excite neurons through universal intramembrane mechanoelectrical effects. *Phys. Rev. X ***8**, 011043 (2018).

[35] Shapiro, M. G., Homma, K., Villarreal, S., Richter, C.-P. & Bezanilla, F. Infrared light excites cells by changing their electrical capacitance. *Nat. Commun. ***3**, 736 (2012). 22415827 10.1038/ncomms1742 PMC3316879

[36] Tolstykh, G. P., Olsovsky, C. A., Ibey, B. L. & Beier, H. T. Ryanodine and ip3 receptor-mediated calcium signaling play a pivotal role in neurological infrared laser modulation. *Neurophotonics ***4**, 025001–025001 (2017). 28413806 10.1117/1.NPh.4.2.025001 PMC5381754

[37] Moreau, D. *et al.* Infrared neural stimulation induces intracellular ca2+ release mediated by phospholipase c. *J. Biophotonics*** 11**, e201700020 (2018). 10.1002/jbio.201700020 28700117

[38] Barrett, J. N. *et al.* Pulsed infrared releases ca2+ from the endoplasmic reticulum of cultured spiral ganglion neurons. *J. Neurophysiol. ***120**, 509–524 (2018). 29668377 10.1152/jn.00740.2017 PMC6139448

[39] Barrett, J. N., Barrett, E. F. & Rajguru, S. M. Mitochondrial responses to intracellular ca2+ release following infrared stimulation. *J. Neurophysiol.*** 129**, 700–716 (2023). 36752512 10.1152/jn.00293.2022 PMC10026987

[40] Ganguly, M., Jenkins, M. W., Jansen, E. D. & Chiel, H. J. Thermal block of action potentials is primarily due to voltage-dependent potassium currents: a modeling study. *J. Neural Eng. ***16**, 036020 (2019). 30909171 10.1088/1741-2552/ab131b PMC11190670

[41] Ford, J. B. *et al.* Optimizing thermal block length during infrared neural inhibition to minimize temperature thresholds. *J. Neural Eng.*** 18**, 056016 (2021). 10.1088/1741-2552/abf00d PMC11189657 33735846

[42] Ford, J. B. *et al.* Identifying the role of block length in neural heat block to reduce temperatures during infrared neural inhibition. *Lasers Surg. Med.*** 52**, 259–275 (2020). 31347188 10.1002/lsm.23139 PMC6981060

[43] Zhuo, J. *et al.* Isotonic ion replacement can lower the threshold for selective infrared neural inhibition. *Neurophotonics*** 8**, 015005–015005 (2021). 33628860 10.1117/1.NPh.8.1.015005 PMC7893321

[44] Borrachero-Conejo, A. I. *et al.* Stimulation of water and calcium dynamics in astrocytes with pulsed infrared light. *FASEB J.*** 34**, 6539–6553 (2020). 32202681 10.1096/fj.201903049R

[45] Jenkins, J. L. *et al.* Laser-induced heating modulates microglial calcium signaling. In *Optical Techniques in Neurosurgery, Neurophotonics, and Optogenetics*, vol. 11629, 1162913 (SPIE, 2021).

[46] Siracusa, R., Fusco, R. & Cuzzocrea, S. Astrocytes: role and functions in brain pathologies. *Front. Pharmacol.*** 10**, 479091 (2019). 10.3389/fphar.2019.01114 PMC6777416 31611796

[47] Bos, R. *et al.* Kv1. 2 channels promote nonlinear spiking motoneurons for powering up locomotion. *Cell Rep. ***22**, 3315–3327 (2018). 29562186 10.1016/j.celrep.2018.02.093 PMC5907934

[48] Davoudi, N., Estrada, H., Özbek, A., Shoham, S. & Razansky, D. Model-based correction of rapid thermal confounds in fluorescence neuroimaging of targeted perturbation. *Neurophotonics*** 11**, 014413–014413 (2024). 38371339 10.1117/1.NPh.11.1.014413 PMC10871046

[49] Fu, P. *et al.* Two-photon imaging of excitatory and inhibitory neural response to infrared neural stimulation. *Neurophotonics*** 11**, 025003–025003 (2024). 38800606 10.1117/1.NPh.11.2.025003 PMC11125280

[50] Chernov, M. M., Chen, G. & Roe, A. W. Histological assessment of thermal damage in the brain following infrared neural stimulation. *Brain Stimul.*** 7**, 476–482 (2014). 24529644 10.1016/j.brs.2014.01.006 PMC4011932

[51] Kiehn, O. Decoding the organization of spinal circuits that control locomotion. *Nat. Rev. Neurosci.*** 17**, 224–238 (2016). 26935168 10.1038/nrn.2016.9 PMC4844028

[52] Kronschläger, M. T. *et al.* Lamina-specific properties of spinal astrocytes. *Glia*** 69**, 1749–1766 (2021). 33694249 10.1002/glia.23990 PMC8252791

[53] Srinivasan, R. *et al.* New transgenic mouse lines for selectively targeting astrocytes and studying calcium signals in astrocyte processes in situ and in vivo. *Neuron*** 92**, 1181–1195 (2016). 27939582 10.1016/j.neuron.2016.11.030 PMC5403514

[54] Chai, H. *et al.* Neural circuit-specialized astrocytes: transcriptomic, proteomic, morphological, and functional evidence. *Neuron*** 95**, 531–549 (2017). 28712653 10.1016/j.neuron.2017.06.029 PMC5811312

[55] Tazerart, S., Vinay, L. & Brocard, F. The persistent sodium current generates pacemaker activities in the central pattern generator for locomotion and regulates the locomotor rhythm. *J. Neurosci.*** 28**, 8577–8589 (2008). 18716217 10.1523/JNEUROSCI.1437-08.2008 PMC6671046

[56] Benham, C. D., Gunthorpe, M. J. & Davis, J. B. Trpv channels as temperature sensors. *Cell Calcium*** 33**, 479–487 (2003). 12765693 10.1016/s0143-4160(03)00063-0

[57] Voets, T. *et al.* The principle of temperature-dependent gating in cold-and heat-sensitive trp channels. *Nature*** 430**, 748–754 (2004). 15306801 10.1038/nature02732

[58] Benfenati, V. *et al.* Expression and functional characterization of transient receptor potential vanilloid-related channel 4 (trpv4) in rat cortical astrocytes. *Neuroscience*** 148**, 876–892 (2007). 17719182 10.1016/j.neuroscience.2007.06.039

[59] Butenko, O. *et al.* The increased activity of trpv4 channel in the astrocytes of the adult rat hippocampus after cerebral hypoxia/ischemia. *PLoS ONE ***7**, e39959 (2012). 22761937 10.1371/journal.pone.0039959 PMC3384594

[60] Shibasaki, K. Trpv4 activation by thermal and mechanical stimuli in disease progression. *Lab. Invest. ***100**, 218–223 (2020). 31896814 10.1038/s41374-019-0362-2

[61] Güler, A. D. *et al.* Heat-evoked activation of the ion channel, trpv4. *J. Neurosci. ***22**, 6408–6414 (2002). 12151520 10.1523/JNEUROSCI.22-15-06408.2002 PMC6758176

[62] Nicotera, P., Bellomo, G. & Orrenius, S. The role of calcium in cell killing. *Chem. Res. Toxicol. ***3**, 484–494 (1990). 2103319 10.1021/tx00018a001

[63] Verkhratsky, A., Untiet, V. & Rose, C. R. Ionic signalling in astroglia beyond calcium. *J. Physiol.*** 598**, 1655–1670 (2020). 30734296 10.1113/JP277478

[64] Herrero, J. F., Laird, J. M. & Lopez-Garcia, J. A. Wind-up of spinal cord neurones and pain sensation: much ado about something?. *Prog. Neurobiol. ***61**, 169–203 (2000). 10704997 10.1016/s0301-0082(99)00051-9

[65] Hachisuka, J. *et al.* Wind-up in lamina i spinoparabrachial neurons: a role for reverberatory circuits. *Pain*** 159**, 1484–1493 (2018). 29578943 10.1097/j.pain.0000000000001229 PMC6053328

[66] Dickenson, A. H. A cure for wind up: Nmda receptor antagonists as potential analgesics. *Trends Pharmacol. Sci. ***11**, 307–309 (1990). 2168102 10.1016/0165-6147(90)90228-z

[67] Davies, S. N. & Lodge, D. Evidence for involvement ofn-methylaspartate receptors in ‘wind-up’of class 2 neurones in the dorsal horn of the rat. *Brain Res. ***424**, 402–406 (1987). 2823998 10.1016/0006-8993(87)91487-9

[68] Thompson, S., King, A. & Woolf, C. Activity-dependent changes in rat ventral horn neurons in vitro; summation of prolonged afferent evoked postsynaptic depolarizations produce a d-2-amino-5-phosphonovaleric acid sensitive windup. *Eur. J. Neurosci.*** 2**, 638–649 (1990). 12106298 10.1111/j.1460-9568.1990.tb00453.x

[69] Woolf, C. J. & Thompson, S. W. The induction and maintenance of central sensitization is dependent onn-methyl-d-aspartic acid receptor activation; implications for the treatment of post-injury pain hypersensitivity states. *Pain*** 44**, 293–299 (1991). 1828878 10.1016/0304-3959(91)90100-C

[70] Mendell, L. M. The path to discovery of windup and central sensitization. *Front. Pain Res. ***3**, 833104 (2022). 10.3389/fpain.2022.833104 PMC8915729 35295805

[71] Talpalar, A. E. *et al.* Dual-mode operation of neuronal networks involved in left-right alternation. *Nature ***500**, 85–88 (2013). 23812590 10.1038/nature12286

[72] Barbay, T. *et al.* Astrocytic kir4. 1 channels regulate locomotion by orchestrating neuronal rhythmicity in the spinal network. *Glia ***71**, 1259–1277 (2023). 36645018 10.1002/glia.24337

[73] Little, S. & Brown, P. The functional role of beta oscillations in parkinson’s disease. *Parkinson. Relat. Disord. ***20**, S44–S48 (2014). 10.1016/S1353-8020(13)70013-0 24262186

[74] Bahadori-Jahromi, F., Salehi, S., Madadi Asl, M. & Valizadeh, A. Efficient suppression of parkinsonian beta oscillations in a closed-loop model of deep brain stimulation with amplitude modulation. *Front. Hum. Neurosci. ***16**, 1013155 (2023). 36776221 10.3389/fnhum.2022.1013155 PMC9908610

[75] Jafari, Z., Kolb, B. E. & Mohajerani, M. H. Neural oscillations and brain stimulation in alzheimer’s disease. *Prog. Neurobiol.*** 194**, 101878 (2020). 32615147 10.1016/j.pneurobio.2020.101878

[76] Luppi, J. J. *et al.* Oscillatory activity of the hippocampus in prodromal alzheimer’s disease: a source-space magnetoencephalography study. *J. Alzheimers Dis.*** 87**, 317–333 (2022). 35311705 10.3233/JAD-215464 PMC9198749

[77] Traikapi, A. & Konstantinou, N. Gamma oscillations in alzheimer’s disease and their potential therapeutic role. *Front. Syst. Neurosci.*** 15**, 782399 (2021). 34966263 10.3389/fnsys.2021.782399 PMC8710538

[78] Horváth, Á. C. *et al.* Histological and electrophysiological evidence on the safe operation of a sharp-tip multimodal optrode during infrared neuromodulation of the rat cortex. *Sci. Rep.*** 12**, 11434 (2022). 35794160 10.1038/s41598-022-15367-4 PMC9259743

[79] Horváth, Á. C. *et al.* Infrared neural stimulation and inhibition using an implantable silicon photonic microdevice. *Microsyst. Nanoeng. ***6**, 44 (2020). 34567656 10.1038/s41378-020-0153-3 PMC8433474

[80] Meneghetti, M. et al. Soft monolithic infrared neural interface for simultaneous neurostimulation and electrophysiology. *Light: Sci. Appl.*** 12**, 127 (2023). 10.1038/s41377-023-01164-9 PMC10209158 37225682

[81] Chernov, M. M., Friedman, R. M. & Roe, A. W. Fiberoptic array for multiple channel infrared neural stimulation of the brain. *Neurophotonics ***8**, 025005–025005 (2021). 33898637 10.1117/1.NPh.8.2.025005 PMC8062107

[82] Coventry, B. S., Lawlor, G. L., Bagnati, C. B., Krogmeier, C. & Bartlett, E. L. Characterization and closed-loop control of infrared thalamocortical stimulation produces spatially constrained single-unit responses. *PNAS Nexus* page 082 (2024). 10.1093/pnasnexus/pgae082 PMC11079674 38725532

