# Supplementary material for: Infrared neuroglial modulation of spinal locomotor networks
Source: Sci Rep. 2024 Sep 27;14:22282. doi: 10.1038/s41598-024-73577-4 (PMC11437012; doi:10.1038/s41598-024-73577-4)
Supplement: Supplementary file 3 — Supplementary Information 3. [file 41598_2024_73577_MOESM3_ESM.pdf]

Supplementary Information

Supplementary video 1: Representative example of infrared-induced damage in astrocytes (green) and neurons (red) induced with a single infrared pulse train at 0.72 $J.cm^{-2}$ showed by sustained increase of calcium fluorescence signals suggesting immediate thermal damage and cell toxicity. (Video accelerated x2).

Supplementary video 2: Representative example of infrared-induced calcium signals in astrocytes (green) and neurons (red) induced with propagation-like behavior observed in astrocytic calcium signaling triggered by a single infrared pulse train at 0.58 $J.cm^{-2}$. (Video accelerated x2).
